# Percutaneous endoscopic lumbar discectomy as a treatment for lumbar disc herniation linked with posterior ring apophysis separation

**DOI:** 10.3389/fsurg.2022.1072444

**Published:** 2023-01-06

**Authors:** Ran Li, Hongyou Zhou, Hao Han, Dongming Fu, Zihao Zhan, Bin Meng

**Affiliations:** Department of Orthopedics, The First Affiliated Hospital of Soochow University, Suzhou, China

**Keywords:** minimally invasive, spinal endoscopy, percutaneous endoscopic lumbar discectomy, lumbar disc herniation, posterior ring apophysis separation, clinical efficacy

## Abstract

**Background:**

Lumbar disc herniation (LDH) linked with posterior ring apophysis separation (PRAS) is a rare and distinct subset of disc herniation. Few studies have evaluated the clinical efficacy of percutaneous endoscopic lumbar discectomy (PELD), which is a procedure used to treat LDH linked with PRAS.

**Objectives:**

To evaluate the clinical efficacy and safety of PELD in the treatment of LDH linked with PRAS.

**Methods:**

Patients who met inclusion criteria (*n* = 67; 40 males and 27 females) underwent PELD. General and operation-related information and perioperative complications of the patients were recorded. Clinical efficacy was measured using the Visual Analog Scale (VAS) and Oswestry Disability Index (ODI) during the follow-up period.

**Results:**

The mean operation time was 118.04 ± 19.31 min and the mean blood loss was 22.84 ± 15.89 ml. The VAS and ODI scores continued to improve immediately after the surgery to the last follow-up. Four patients experienced postoperative complications i.e., herniation recurrences. The conditions of the patients with the complications improved after treatment.

**Conclusions:**

PELD has reliable efficacy and safety in the treatment of LDH linked with PRAS.

## Introduction

Posterior ring apophysis separation (PRAS) refers to the discovery of a bone fragment near the posterior horn of the lumbar vertebrae. The size and shape of the free bone fragment are almost consistent with the bone defect at the posterior edge of the vertebral body, and there is marked bone sclerosis at the same site of the defect, thereby indicating that the bone fragment originates from the vertebral body (1). PRAS tends to occur in parallel with lumbar disc herniation (LDH) whenever LDH is detected. The symptoms of LDH linked with PRAS are chronic low back pain and leg pain, which are comparable to those of a simple lumbar disc herniation or lumbar spinal stenosis; therefore, these symptoms are often missed or clinically ignored (2). For patients with radiculopathy or cauda equina nerve damage whose conditions do not improve after receiving conservative care, surgical treatment should be performed as soon as possible. Open surgery is the standard course of treatment, which adequately decompresses the nerve roots, but the surgical trauma is high and the stability of the lumbar spine is greatly affected (3). PRAS frequently occurs in young and middle-aged adults, and lesions are mostly concentrated in the lower lumbar spine (4). The main goal of the treatment should be to avoid fixation and fusion as much as possible to preserve the natural structure and motor functions of the spine, especially given the high prevalence of PRAS among young adults (5). It is important to pay attention to the issue of surgical trauma and manage iatrogenically aggravated disc degeneration in the surgical and adjacent segments.

Percutaneous endoscopic lumbar discectomy (PELD) technology has advanced markedly since, in 1996, Kambin attempted to use arthroscopy to perform a discectomy (6). PELD is classified into two types, percutaneous endoscopic interlaminar discectomy (PEID) and percutaneous endoscopic transforaminal discectomy (PETD), in accordance with the surgical approach (7). PELD has several benefits over traditional open discectomy and fusion surgery, including minimal invasion, minimal blood loss, quick recovery, etc (8–11). To the best of our knowledge, there have been only few studies published regarding the efficacy of PELD in treating LDH linked with PRAS. This study aims to analyze the follow-up data of patients with LDH linked with PRAS and explore the technical aspects of PELD to obtain greater clinical efficacy and lower incidences of complications.

## Materials and methods

### General information

The clinical information of the patients who underwent PELD as a treatment for LDH linked with PRAS was reviewed; the patients were followed up from January 2015 to October 2020. The inclusion criteria were: (1) symptoms of typical unilateral low back and leg pain along with numbness, (2) imaging findings that show disc herniation with the posterior edge of the vertebral body disconnected and the corresponding segment nerve compressed, (3) conservative treatment for at least 3 months, including bed rest and nonsteroidal anti-inflammatory drugs, which have limited clinical effects. The exclusion criteria were: (1) previous surgical history of treatment for spinal stenosis and motion instability, (2) other spinal disorders such as ankylosing spondylitis and spinal tumors and fractures, (3) dementia, intellectual disability, and drug abuse.

### Surgical methods

The surgery was performed by four senior surgeons, but they followed the same technical principles. The surgical method was opted based on different surgical segments and the type of disc herniation. PEID was routinely performed on L_4-5_ and L_5_-S_1_ segments, and PETD was used to treat cases with extreme lateral disc herniation.(1) PEID: The patients were placed in a prone position on a Wilson table and treated under general anesthesia. An incision was made 1 cm lateral to the posterior midline on the level of the treated intervertebral space. A dilator was bluntly put through the skin into the subcutaneous tissue and the muscles. The working channel was then inserted when the dilator reached the ligamentum flavum. The ligamentum flavum was dissected and separated, and epidural space was exposed. The herniated disc was removed using straight and nucleus pulposus forceps. The bone fragments were fully removed using a microscopical trephine, grinding drill, and bone knife. The nerve roots were probed to ensure adequate decompression. The tunnel was exited and the wound was closed.

(2) PETD: The patients were placed in the prone position with hip and knee joint flexion, and local anesthesia was administered. Under the guidance of fluoroscopy, a puncture needle was inserted into the upper posterior area of the caudal vertebra *via* the zygapophyseal joint. Then, the dilator and reamer protector were placed through a guiding wire. The tips of the dilator and reamer protector were localized near the medial side of the pedicular on an anteroposterior (AP) view and at the upper posterior area of the caudal vertebra on a lateral view. A 7.5 mm-reamer was inserted and foraminoplasty was performed using the tip of the reamer advancing to the medial side of the pedicular on the AP view under the fluoroscopy. The remaining steps were the same as PEID.

Second-generation cephalosporin or clindamycin was prophylactically used during the surgery and not used after the surgery, except under special circumstances. There was no need of postoperative radiography. All the patients were encouraged to exercise with the assistance of a lumbar brace on the first postoperative day and were mostly discharged on the second postoperative day with further follow-up at the outpatient clinic.

### Clinical outcomes

Outcome indicators were determined through medical record review. Clinical outcomes were collected from the patients' reported outcomes. Hospitalization data were reviewed to identify any intraoperative complications. Intraoperative blood loss was assessed by the surgeons according to the specific intraoperative bleeding conditions, operation time, and the patients' hemoglobin level before and after the surgery. Postoperative complications were defined as any adverse events occurring within 30 days after the surgery. The date of the last outpatient appointment in the Department of Orthopedics was defined as the date of the last follow-up visit. Perioperative complications and their treatment were also recorded and analyzed. The Visual Analog Scale (VAS) was applied to assess the extent of back pain and radicular leg pain. The Oswestry Disability Index (ODI) was employed to evaluate function and life quality. Different data were recorded on five time points, including pre-operation, post-operation, 1 month after surgery, 3 months after surgery, and the last follow-up. Postoperative evaluation was conducted between two time points, one was the time when the patients were able to get out of bed with the lumbar brace, and another was the time when the patients were discharged.

Surgical results were graded as “excellent” when there was no pain and no limitations for any activity, “good” when back pain or leg pain due to any strenuous activity was occasionally reported, “fair” when the symptoms improved after the surgery but recurrent or residual pain led to restricted activities, and “poor” when the symptoms did not improve or worsened after the surgery.

### Statistical analysis

All quantitative data were presented as mean ± standard deviation and analyzed using SPSS 24.0 (SPSS Inc., Chicago, IL, United States). The perioperative clinical outcomes were compared using the paired *t*-test. The difference in the values obtained was considered statistically significant when *p* < 0.05.

## Results

### General information

**Figure 1:**
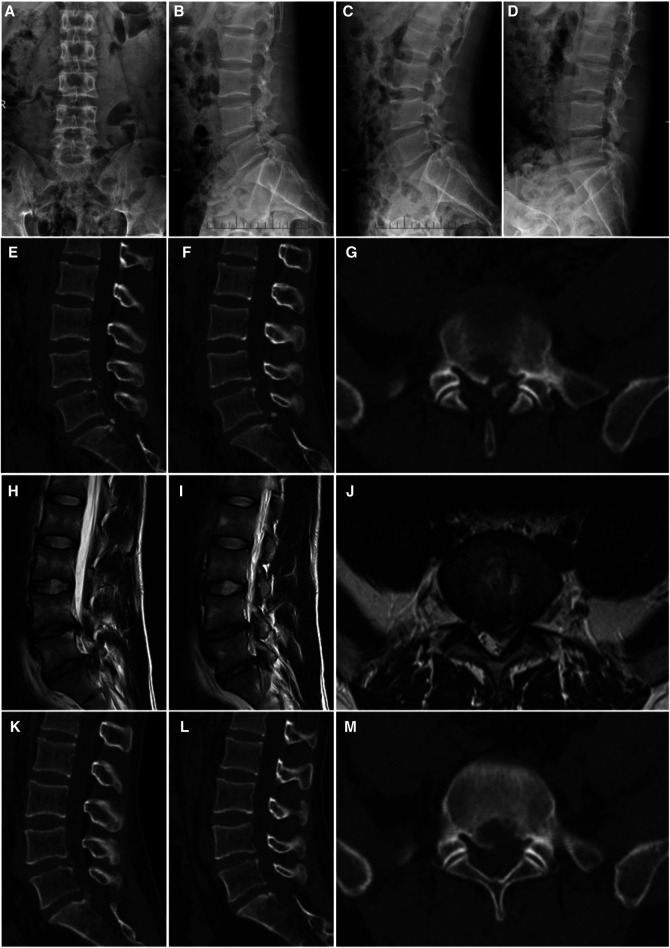
A 26-year-old man with LDH linked with PRAS was treated with PETD; the main symptoms were low back pain with radiating pain in the left lower limb. (**A,B**) anterior and lateral X-ray images of the lumbar, (**C,D**) dynamic X-ray images of the lumbar, (**E–G**) CT scan images of the lower lumbar spine: the detached bone fragment is shown to protrude into the posterior margin of the vertebral body, (**H–J**) MRI of the lumbar spine: the imaging findings were consistent with that of the CT scans, and some of the discs are shown to protrude laterally, (**K–M**) the CT scan images show that the bone fragment pressing the nerve on the patient's left side has been removed.

**Figure 2:**
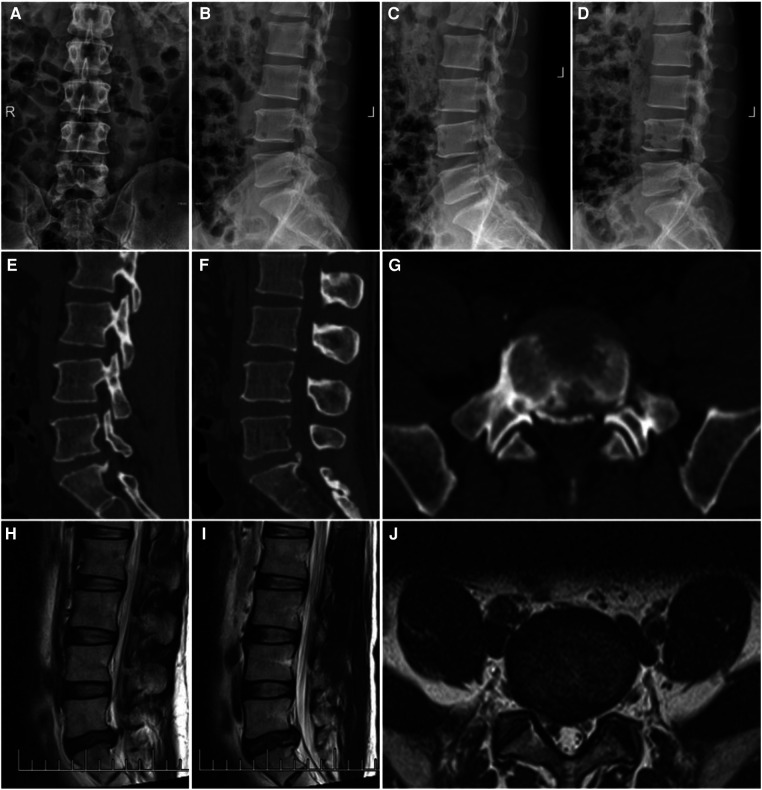
A 44-year-old man with LDH linked with PRAS was treated with PEID; the symptoms were mainly pain in the right lower limb. (**A,B**) CT scan of the lower lumbar spine: the detached bone fragment is shown to protrude into the posterior margin of the vertebral body and (**C,D**) MRI of the lumbar spine: the imaging findings were consistent with that of the CT scan.

**Figure 3:**
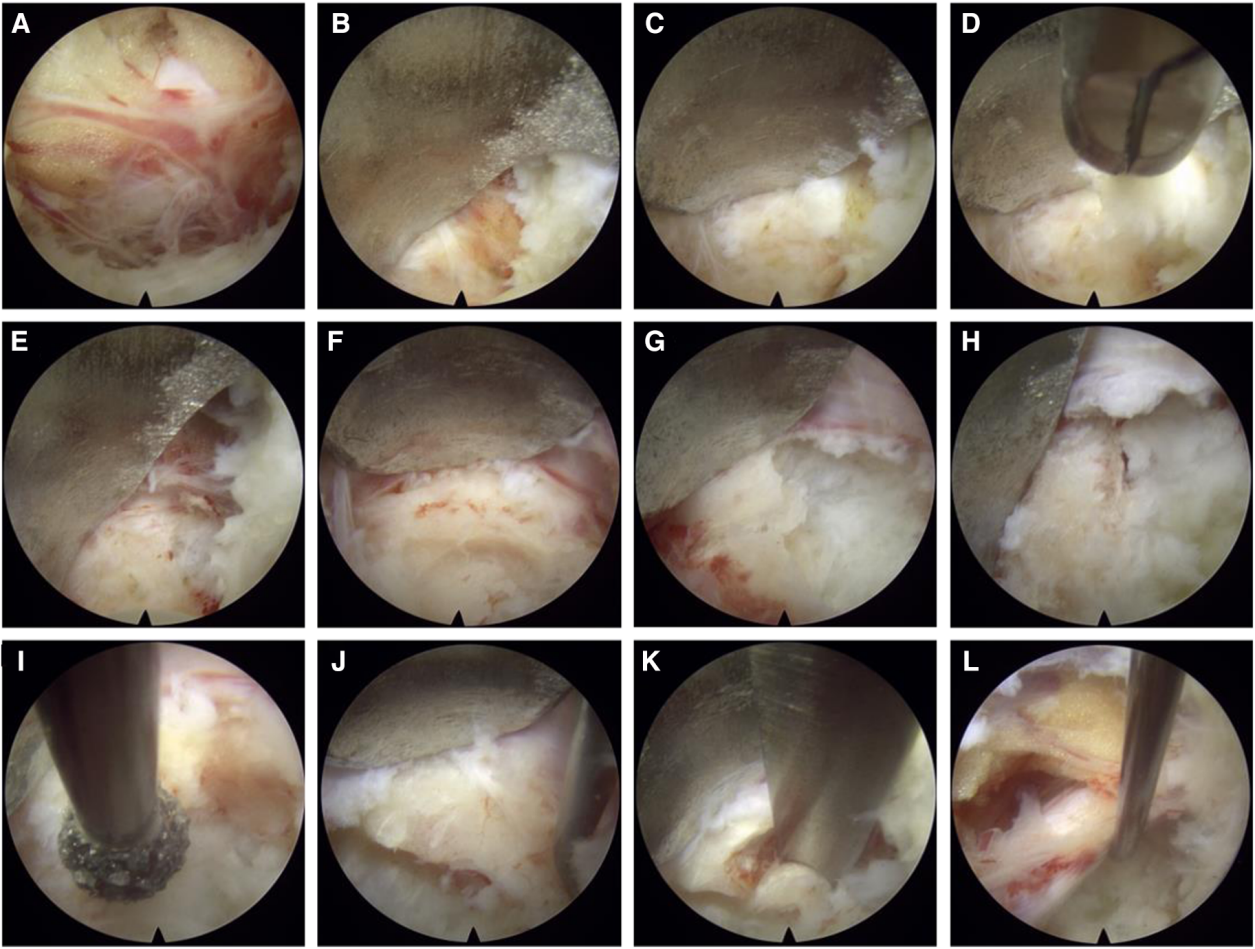
Imaging during surgical procedures: (**A**) exposure of nerve roots; (**B**) the nerve root is shown to be dissected, and the bone behind it and the protruding fibrous annulus are exposed; (**C,D**) annulus fibrosus and nucleus pulposus are shown to be removed with luminal forceps; (**E–K**) after the annulus fibrosis is shown to be removed, and the bone is also shown to be removed using trephine, grinding drill, and endoscopic bone knife; (**I**) nerve root relaxation is shown after decompression.

**Figure 4:**
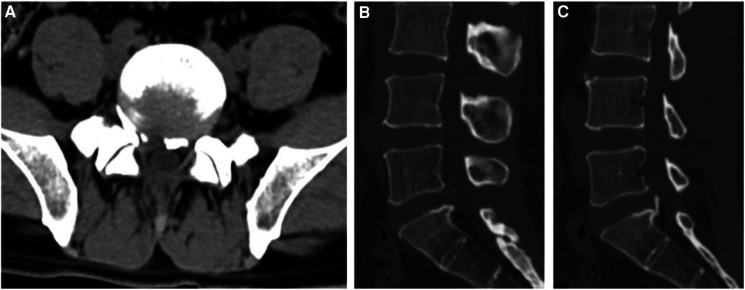
Postoperative CT images: (**A**) transverse CT image of the patient in figure 2 after the operation shows that the bone fragment that compressed the nerve root on the right side has been removed, (**B**) a sagittal CT scan shows that the right bone fragment has been completely removed, (**C**) the sagittal CT scan shows that the left bone fragment is firmly connected to the vertebral body and is not resected during the operation.

The characteristics of the patients are summarized in Table 1. A total of 67 patients, including 40 males and 27 females, met the inclusion criteria and were recorded and followed up. The mean age of the patients was 40.31 ± 7.39 years (26∼55 years) and the mean follow-up duration was 31.15 ± 14.17 months (12∼72 months). Injuries directly related to the onset of new symptoms were observed in 16 patients (23.88%) and were attributed to falls or motor vehicle accidents. Typical cases are shown in Figures 1–4.

**Table 1:** Demographical and surgical information.

Patient characteristics		Outcomes
Number	67	
Gender (males/females)	40/27	
Age (year)	40.31 ± 7.39	
No. of patients with an injury as the cause of the disease	16	
Follow-up time (month)	31.15 ± 14.17	
Segments	L_4-5_	29
	L_5_-S_1_	38
Operation duration (min)	118.04 ± 19.31	
Blood loss (ml)	22.84 ± 15.89	

### Surgical information

All the patients underwent a unilateral approach on the symptomatic side and under general anesthesia. The most common segment operated on was L_5_-S_1_ followed by L_4-5_. Transverse disc herniation occurred in 49 patients, out of which 26 patients had their left side involved. Central disc herniation occurred in 18 patients. The S_1_ superior endplate was the most common site (39 cases), followed by the L_5_ superior endplate (23 cases). All the patients were classified and staged according to Table 2 (2). Type I occurred in 43 patients and type II in 24 patients; 35 patients had stage A and 32 had stage B (Table 3). The mean operation duration was 118.04 ± 19.31 min, and the mean blood loss was 22.84 ± 15.89 ml. There was no anesthesia-related complication in all the patients. In this study, 27 patients had complete bone fragment resection and 40 patients had only partial bone fragment resection.

**Table 2:** Type and stage of PRAS.

Type and stage	Description
Type I	The bone fragment is still articulated to the vertebral body, and the bone fragment adjacent to the disc is partially separated from the posterior edge of the vertebral body.
Type II	The bone fragment is completely separated from the posterior margin of the vertebral body.
Stage A	The disc is displaced to the posterior margin of the bone fragment.
Stage B	The disc is displaced beyond the posterior margin of the bone fragment.

**Table 3:** Postoperative type and stage.

Vertebra	No. of cases				
	Total	Type I	Type II	Stage A	Stage B
L4					
Inferior endplate	3	1	2	2	1
L5					
Superior endplate	23	15	8	12	11
Inferior endplate	2	1	1	1	1
S1					
Superior endplate	39	26	13	20	19
Total	67	43	24	35	32

### Clinical outcomes

All the patients underwent the surgery successfully and were followed up as mentioned in Table 4. The mean VAS (low back), VAS (leg), and ODI scores before the surgery were 7.82 ± 1.14, 6.01 ± 0.77, and 78.96 ± 4.83%, respectively. The postoperative VAS and ODI scores of the patients were considerably lower than the preoperative scores of the patients. There was no remarkable difference between the scores of the two groups during the entire follow-up period. According to the modified Macnab criteria, there were 60 excellent cases, 3 good cases, and 4 fair cases; the rate of excellent or good outcomes was 94.03%. No postoperative neurological deficit or deterioration of preoperative functions was observed.

**Table 4:** Clinical outcomes before and after surgery.

	Pre-operation	Post-operation	1 month	3 months	Last follow-up
VAS (low back)	7.82 ± 1.14	1.36 ± 1.14*	1.52 ± 1.20*	1.85 ± 1.36*	2.51 ± 1.74*
VAS (leg)	6.01 ± 0.77	1.76 ± 0.63*	1.54 ± 0.61*	1.45 ± 0.56*	2.06 ± 1.13*
ODI (%)	78.96 ± 4.83	18.75 ± 8.17*	19.13 ± 7.70*	21.34 ± 5.46*	23.58 ± 16.07*
MacNab	Rate of excellent or good: 94.03%				

*p < 0.05 compared with preoperative data.

### Complications

Out of the 67 patients, 4 suffered disc herniation recurrence in the same segment. Out of that, two patients got relieved after the conservative therapy, one patient underwent endoscopic revision surgery, and the other patient underwent posterior lumbar intervertebral fusion surgery. All the patients with recurrent disc herniation had considerable improvement in their symptoms and no obvious back pain or radicular pain remained.

## Discussion

LDH linked with PRAS is a rare and distinct subset of disc herniation. Because of the small size and unfamiliarity of the bone fragments in this condition, they are often missed on radiographs and confused mainly with calcified protrusion of the posterior longitudinal ligament or intervertebral disc and dorsal osteophyte degeneration. PRAS accompanied by LDH tends to be more common in young adults and occurs mainly in adolescents who have a history of traumatic episodes or performing repeated vigorous exercises. Many studies have suggested that trauma is considered to be the main etiology for the development of PRAS in adolescents, and chronic vigorous activity affecting the lumbosacral spine may be the main event leading to the onset of the disease symptoms in adults (12). The symptoms of low back pain are often aggravated after the trauma and are accompanied by radicular pain that radiates into the lower extremity directly along the course of a specific spinal nerve root. The clinical manifestations and signs of LDH linked with PRAS are similar to those of lumbar disc herniation and lumbar spinal stenosis, and therefore are difficult to distinguish. The conventional lumbar X-ray does not clearly show the herniated discs. However, a CT scan can clearly show the shape, location, and complexity of the herniated discs and the broken bone mass of the posterior edge of the vertebral body, this is the most effective way to diagnose the disease. Furthermore, an MRI scan can visually and clearly show the location, size, and degree of the nerve compressed by the herniated discs. PRAS is mostly concentrated in the lower lumbar spine; L_5_-S_1_ is the most common site of the posterior edge of the vertebral body, followed by L_4-5_. In the present study, there were 38 patients with herniation at the L_5_-S_1_ segment, 29 with herniation at the L_4_-_5_ segment. The most common site is the posterior superior edge of S_1_, followed by the posterior superior edge of L_5_, and the posterior inferior edge of L_4_. From a functional point of view, probably because the pressure on the superior endplate is the greatest, all the superior endplates are affected more than the inferior endplates (13).

Once LDH linked with PRAS is diagnosed, surgical treatment is often required. According to previously reported large or small incision fenestration procedures, half or total laminar decompression surgery has certain disadvantages such as severe surgical trauma and damage to the stability of the lumbar spine (3). In order to avoid postoperative segmental instability, fixation and fusion therapies are used, which lead to issues such as accelerated adjacent segment degeneration and adjacent vertebral diseases in the long term. With the increasing advancement in percutaneous spinal endoscopy, surgical indications have gradually expanded, and it has been used in the treatment of various types of LDH and spinal stenosis (6, 14–17). PELD has advantages such as it causes minimal extensive dissection of paravertebral muscles, blood loss, and tissue damage. Moreover, it causes minimal damage to the normal structure of the spine, completely retains the middle and posterior column structure, does not affect the stability of the spine, and reduces the incidence of postoperative complications such as lumbar spondylolisthesis and low back pain (11). The operation time for PELD, when compared to that of traditional open surgery, is considerably less. In addition, the recovery time is less, a patient can wear the lumbar brace to get out of bed on the day after surgery, the second day the patient is discharged, and returns to normal work and life 1 month after the surgery. This surgery can reduce the pain experienced by patients and reduce the cost of hospitalization.

In this study, the patients recovered well from the pain after undergoing spinal endoscopic techniques. To obtain a safe and effective course of treatment for LDH linked with PRAS, we should pay great attention to perioperative details. With the help of complete preparation before the operation, accurate analysis of the scan reports and identification of clear symptoms of the responsible lesion segmentation and the size and direction of the disc herniation and broken bone block, intraoperative decompression can be effective to achieve the expected surgical outcome. According to the imaging findings, an appropriate surgical approach was adopted. PRAS is often accompanied by LDH; therefore, the surgical plan can be designed according to the location of the disc herniation. If the disc herniation is mainly located within the lateral recess near the midline, the interlaminar approach is used. If the disc herniation is mainly located beyond the lateral recess to the extramarginal region, the foraminal approach is used. Due to the limited operation space under the endoscope, the operation should be performed gently, and the bleeding should be promptly stopped by bipolar electrocoagulation. A clear surgical field of view is the basic requirement for the surgery, and a clear visual field should be maintained to distinguish the herniation structure and avoid accidental injury of the dural sac and nerve roots. During the operation, the protruding annulus fibrosus and nucleus pulposus are excised to reduce tension, and the working channel is slightly adjusted. The working channel could be appropriately tilted to increase the exposure range. Combined with the use of endoscopic trephine, grinding drill, and endoscopic bone knife, the bone block of the posterior edge of the vertebral body could be removed properly. Complete resection of the bone fragment is not emphasized in the operation. If the bone block is stable and does not cause compression, complete resection can be avoided to prevent nerve root injury and aggravation of symptoms due to the surgery.

Simultaneous removal of epiphyseal fragments during discectomy may be a controversial approach. Most scholars believe that the bone mass and intervertebral disc tissue should be removed completely after vertebral body resection to completely relieve the compression of the protrusion on the nerve root or cauda equina nerve (1, 18–20). On the contrary, other researchers believe that the initial factor involved in the symptoms caused by the rupture of the posterior edge of the lumbar vertebrae is disc herniation; therefore, during the operation, the disc must be removed, thereby the cause of LDH, which is disc herniation, will also be removed. Furthermore, if the posterior fracture of the bone fragment, did not cause organic compression and stenosis, there is no need for complete resection. Shirado et al. conducted a prospective study on 32 patients: 11 of them underwent discectomy and posterior margin bone mass resection and 21 underwent only discectomy (21). After follow-up, the two groups of patients were satisfied with the curative effect, indicating that the removal of the severed bone block did not affect the clinical curative effect. Therefore, the researchers believe that if the bone is not free, it does not need to be completely removed in the process to achieve thorough decompression. There is also no clear evidence that the broken posterior bone mass can develop progressively, leading to recompression, and that the unremoved bone mass can lead to chronic low back pain.

We believe that the primary objective of the surgery is to adequately decompress the nerve root and that the removal of the severed bone mass is not always necessary. There are two main types of PRAS. One is proposed by Takata K et al. in 1988: type I, cortical detachment at the posterior margin of the vertebral body; type II, avulsion fractures of the posterior margin of the vertebral body, including cortical and cancellous bones; type III, a bone mass near one side is detached, accompanied by a bone defect (1). The present study takes a different approach as shown in Table 2 (2). We believe that this classification is more suitable for guiding the treatment of PRAS using spinal endoscopic techniques. We suggest that bone fragment in type I PRAS should be excised only after the stability of the residual bone mass is determined after the partial bone mass affecting the nerve root is removed and sufficient decompression of the nerve root is ensured, while the bone fragment in type II PRAS must be excised by default. Although the main cause is the compression of the nerve by the herniated disc, in stage A (Table 2), complete resection is recommended when typical symptoms appear. In stage B (Table 2), excessive resection of the bone mass is not necessary after sufficient decompression and stability of the bone fragment is ensured. It is necessary to preserve as much of the posterior vertebral body and unruptured disc as possible, especially in active adolescents.

In this study, during the operation, the nerve root was often excessively pulled when we wanted to completely remove the broken bone. On the other hand, the use of conventional cavity and nucleus pulposus forceps is difficult to remove hard bone blocks during the operation. Therefore, ring saws, grinding drills, and bone knives are often used. All the surgical equipment greatly increase the possibility of nerve root damage. It is noteworthy that during the process of bone block resection, the cut bone block will be sharp; therefore, the cannula and the sharp bone block edge should be prevented from cutting the nerve root. The short-term postoperative effect on patients who experience such nerve root damage is relatively poor; however, it can be alleviated using conservative treatment with drugs.

In conclusion, this study presents the primary outcome of the evaluation of the efficacy and safety of PELD in the treatment of LDH linked with PRAS. This study involves more cases and a longer follow-up duration as compared to those in previously reported studies. However, this study also has some limitations such as sample size. PELD has great efficacy and safety in the treatment of LDH linked with PRAS in a short follow-up duration. In addition, for further outcomes, a large sample size and multi-center randomized controlled trials are required.

## Data Availability

The raw data supporting the conclusions of this article will be made available by the authors, without undue reservation.
